# Identifying the druggable interactome of EWS-FLI1 reveals MCL-1 dependent differential sensitivities of Ewing sarcoma cells to apoptosis inducers

**DOI:** 10.18632/oncotarget.25760

**Published:** 2018-07-24

**Authors:** Kalliopi Tsafou, Anna Maria Katschnig, Branka Radic-Sarikas, Cornelia Noëlle Mutz, Kristiina Iljin, Raphaela Schwentner, Maximilian O. Kauer, Karin Mühlbacher, Dave N.T. Aryee, David Westergaard, Saija Haapa-Paananen, Vidal Fey, Giulio Superti-Furga, Jeffrey Toretsky, Søren Brunak, Heinrich Kovar

**Affiliations:** ^1^ Disease Systems Biology, Novo Nordisk Foundation Center for Protein Research, Faculty of Health and Medical Sciences, University of Copenhagen, Copenhagen, Denmark; ^2^ CeMM Research Center for Molecular Medicine of the Austrian Academy of Sciences, Vienna, Austria; ^3^ Children’s Cancer Research Institute, St. Anna Kinderkrebsforschung, Vienna, Austria; ^4^ Department of Pediatrics, Medical University of Vienna, Vienna, Austria; ^5^ Medical Biotechnology, VTT Technical Research Centre of Finland, Espoo, Finland; ^6^ Department of Oncology, Georgetown University, Medical Center, Washington, DC, USA; ^7^ Current address: Broad Institute of MIT and Harvard, Cambridge, MA, USA

**Keywords:** high-throughput compound screening, Ewing sarcoma, drug-target network, apoptosis, BCL-2 inhibitors

## Abstract

Ewing sarcoma (EwS) is an aggressive pediatric bone cancer in need of more effective therapies than currently available. Most research into novel targeted therapeutic approaches is focused on the fusion oncogene *EWSR1-FLI1*, which is the genetic hallmark of this disease. In this study, a broad range of 3,325 experimental compounds, among them FDA approved drugs and natural products, were screened for their effect on EwS cell viability depending on EWS-FLI1 expression. In a network-based approach we integrated the results from drug perturbation screens and RNA sequencing, comparing EWS-FLI1-high (normal expression) with EWS-FLI1-low (knockdown) conditions, revealing novel interactions between compounds and EWS-FLI1 associated biological processes. The top candidate list of druggable EWS-FLI1 targets included genes involved in translation, histone modification, microtubule structure, topoisomerase activity as well as apoptosis regulation. We confirmed our *in silico* results using viability and apoptosis assays, underlining the applicability of our integrative and systemic approach. We identified differential sensitivities of Ewing sarcoma cells to BCL-2 family inhibitors dependent on the EWS-FLI1 regulome including altered MCL-1 expression and subcellular localization. This study facilitates the selection of effective targeted approaches for future combinatorial therapies of patients suffering from Ewing sarcoma.

## INTRODUCTION

Ewing sarcoma (EwS) belongs to a family of highly malignant primary tumors, which arise in bone and soft tissues, affecting children and adolescents [1]. The genetic hallmark of EwS is the presence of the chromosomal translocation t(11;22) (q24;q12) that generates the *EWS-FLI1* fusion gene [2]. The resulting fusion protein, which is found in 85% of EwS patients, contains the amino terminal domain of the transcriptional activator EWS, and the carboxyl terminal domain of the DNA binding protein FLI1. EWS-FLI1 is an aberrant transcription factor that both activates and represses expression of hundreds of genes, many of them being crucial for EwS pathogenesis. EWS-FLI1 has been characterized as the “Achilles’ heel” of EwS and an ideal therapeutic target [3, 4]. Recently, the small molecule YK-4-279 was shown to interfere directly with the EWS-FLI1 activity by blocking its interaction with RNA helicase A. An analog of YK-4-279 has now entered clinical trials (NCT02657005) [5]. Exploiting the downstream network of EWS-FLI1 is crucial for the discovery of alternative inhibitory scaffolds.

In this study we used a network-based integrative analysis platform to investigate druggable target gene spectra of EWS-FLI1. Among the top druggable target hits we found compelling evidence for EWS-FLI1-dependent sensitivities to BCL-2 family member inhibitors. Depending on their BCL-2 homology (BH) domains and function, the BCL-2 family of proteins can be classified into three different groups. The pro-apoptototic BCL-2 family members, BAX and BAK, anti-apoptotic memebers BCL-2, MCL-1, BCL-X, BCL-W and BFL-1/A1. The third class of BCL-2 family members consists of the so-called BH-sensitizers BAD, BIK, NOXA, BMF, Harakiri and PUMA [6]. The balance of pro- and antiapoptotic BCL-2 proteins is crucial for cell survival and commonly exploited by cancer cells, which due to their exaggerated metabolism, oncogenic stress and cancer therapy are more primed for cell death [6–8]. Via alternative splicing the long isoform (L) of anti-apopototic BCL-2 family member proteins can be shortened into a pro-apoptotic version (S), such as for MCL-1 (L/S) and BCL-X (L/S), further influencing the balance between pro- and antiapoptotic proteins within a cell [7]. Given the importance of BCL-2 proteins for oncogenic cell survival, several BCL-2 family inhibitors, so called BH3 mimetics, have been developed. Obatoclax (GX-15-070) binds to a broad range of BCL-2 family proteins with low affinity, in a BAX/BAK independent manner. ABT-737 and navitoclax (ABT-263) more specifically inhibit BCL-2 and BCL-X(L) [8] and exhibit greater bioavailability and improved clinical responses. Resistance mechanisms via MCL-1, however, have frequently been reported for these BH3 mimetics [8, 9]. Here, we report major differences of EwS cells in the response to obatoclax and navitoclax or ABT-737, depending on the EWS-FLI1 expression status. Investigation of BCL-2 family member protein expression and their subcellular localization revealed an EWS-FLI1 dependent effect on MCL-1(L) to be at least partially responsible for the differential sensitivities of EwS cells towards navitoclax treatment. The results confirmed our systematic approach and yielded novel insights into the druggable interactome of EwS.

## RESULTS

### Establishing criteria for hit selection

**Figure 1:**
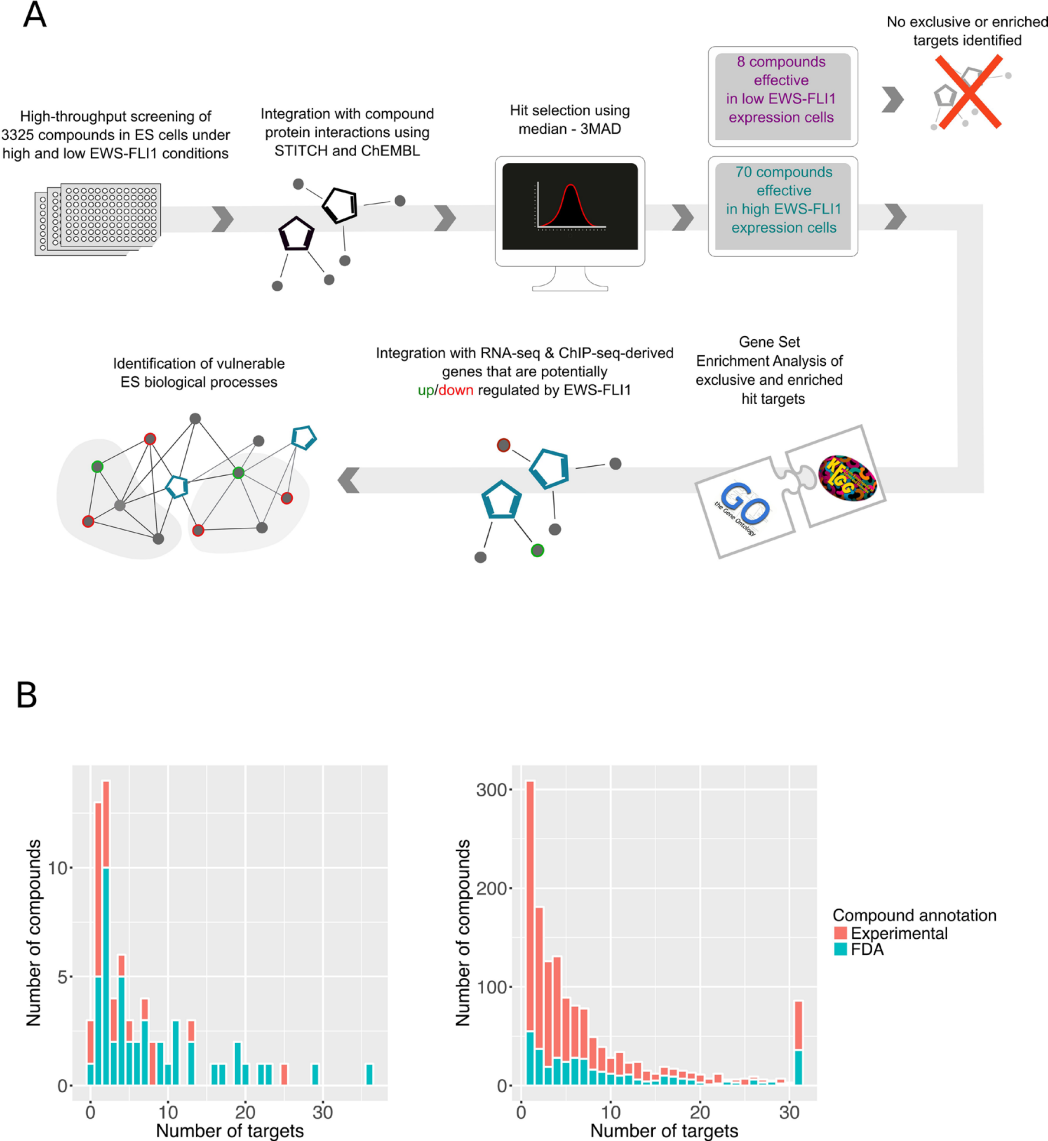
Compound and target discovery (**A**) Schematic representation of our systematic approach developed to identify effective compounds and biological vulnerabilities in EwS. (**B**) Distribution of targets per compound. Left: Target distribution of the effective compounds (hits). The effective compounds with known targets included 23 experimental and 47 FDA approved compounds. FDA approved drugs cover the major percentage of the 70 hits (67%), and the median of the reported targets is 4. Right: Target distribution of the non-effective compounds. The median of targets for the non-effective compounds (with at least one known target) is 5 and the list includes 27% (394) FDA approved compounds.

In this study, we performed a high-throughput phenotypic screen of 3,325 FDA-approved and experimental compounds in an EwS cell line, A673/TR/shEF, where EWS-FLI1 expression can be modulated from high to low via doxycycline (dox)-inducible shRNA expression [10–12] (Workflow: Figure 1A). To identify selective anti-proliferative compounds effective under EWS-FLI1-high and -low conditions, cells were either cultured without dox and exposed to drugs for 72 h (EWS-FLI1-high condition), or pre-treated with dox for 24 hours and then exposed to drugs for 72 hours in the presence of dox (EWS-FLI1-low condition) (Supplementary Figure 1A, Supplementary Tables 1 and 2). To gain insights into the target spectra of all screened compounds (1,515 compounds with reported targets), we used the chemical protein interaction resources ChEMBL [13] and STITCH [14] (Supplementary Table 3). To more specifically study compounds interfering with EWS-FLI1 activity, we furthermore grouped the tested compounds into (i) those that present increased efficacy in EWS-FLI1-high cells and (ii) those that are more potent in EWS-FLI1-low cells. The criterion for assessing increased efficacy was a decrease in viability at a single concentration by at least two-fold for EWS-FLI1-high cells versus EWS-FLI1-low cells for the group (i) and vice versa for the group (ii) (see Materials and Methods & Supplementary Table 1). A third group of compounds reduced cell viability independent of EWS-FLI1 expression, likely by EWS-FLI1-independent mechanisms.

### Compounds that are more effective in EWS-FLI1-high than in EWS-FLI1-low EwS cells

We found 70 out of the 3,325 unique compounds to be more effective in EWS-FLI1-high cells, of them 47 are already FDA approved (Supplementary Table 4). After acquiring the known targets of all the tested compounds, we confirmed that the hit selection was not biased towards the number of compound targets (compound promiscuity). The median number of targets corresponding to the 70 hits was 4, while by comparison 5 targets per compound were represented in the group of non-hits (Figure 1B).

To gain further insight into the mechanisms of action for the selected compound hit list, we extracted (i) the genes that were exclusively targeted by the hit compounds and (ii) the genes that were significantly enriched targets in the list of hit compounds (Fisher’s exact test, *q*-value < 0.05). The two gene sets were combined to form a single candidate gene list for further analysis (Supplementary Table 5). We applied functional annotation gene enrichment using the DAVID tool suite [15], more specifically the Gene Ontology branches “Biological process” and “Molecular function”, as well as the KEGG pathway [16] database to study the assigned functions of our candidate gene list [17].

**Figure 2:**
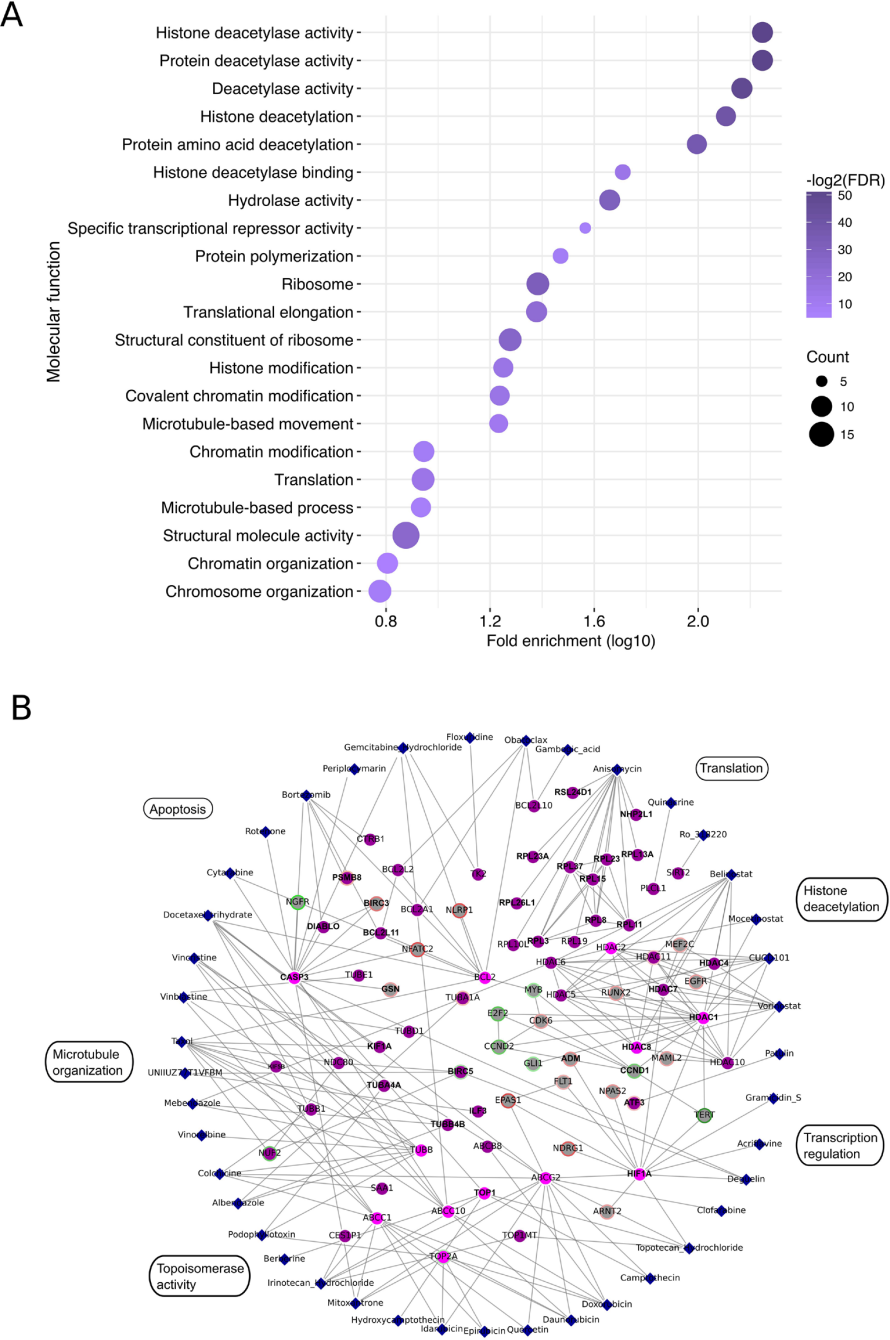
Compound-target interaction network (**A**) Biological processes significantly associated with the exclusive and enriched targets of the potent compounds in EWS-FLI1 high EwS cells. Color scale represents the Benjamini-Hochberg adjusted *p*-value while the circle size is analogous to the number of genes found to be involved in the corresponding terms. Histone deacetylase activity, apoptosis, ribosome biogenesis and topoisomerase activity were among the top processes affected. (**B**) Interactions between potent compounds (blue diamonds) and genes, which were found to be exclusively targeted (purple nodes) or enriched in the list of potent compounds (cyan nodes) in EWS-FLI1-high EwS cells. Genes which increased or decreased in expression upon EWS-FLI1 knockdown (RNA-seq data derived by [10]) are highlighted with red and green halos, respectively. Color intensity indicates degree of regulation. Potential EWS-FLI1 targets according to Chip-seq data derived by [18] are indicated with bold black text.

The druggable candidate gene list contained genes associated with critical cellular functions such as translation (anisomycin), histone modification (HDAC inhibitors belinostat, vorinostat, mocetinostat and CUDC-101), microtubule structure (vincristine, vinblastine and taxol), topoisomerase activity (irinotecan, topotecan, idarubicin and doxorubicin) as well as apoptosis regulation (cytarabine, bortezomib, gambogic acid and obatoclax mesylate) (Figure 2A). Furthermore, members of the ABC transporter family (e.g. ABCC10, ABCC1, ABCG2 and ABCB8), as well as transcription factors such as ATF3, ILF3 and HIF-1a were among the candidate gene sets (Figure 2B and Supplementary Table 6).

To further annotate our candidate set of druggable genes in EwS to EWS-FLI1 expression, we first integrated previously published RNA-seq data from EWS-FLI1-high and -low EwS cells into our analysis [10]. A minimum of 1-fold change in gene expression upon EWS-FLI1 modulation (72 h dox treatment) and a *p*-value cutoff after False Discovery Rate (FDR) adjustment for multiple testing of 0.01 was accepted for further analysis. Next, we combined this information with previously published ChIP-seq-derived EWS-FLI1 direct targets [18]. Using the STRING database [19], interactions between effective compounds and EWS-FLI1 direct and indirect targets were mapped (Figure 2B). Thereby, we found so far unrecognized interactions, such as between HDAC inhibitor hits and the EWS-FLI1 regulated genes *EGFR*, *MEFC2*, *RUNX2* and *CCND1*. A number of compounds were also found to interfere with EWS-FLI1 regulated genes, which are involved in translation and apoptosis. In particular, multiple members of the ribosomal protein family, *BIRC3*, *NLRP1*, *GSN* and *NGFR* were affected by EWS-FLI1 modulation and chemical inhibition (Figure 2B). A list of the compound targets and respective RNA-seq and ChIP-seq-derived annotation is provided in Supplementary Table 3.

### Compounds that are more effective in EWS-FLI1-low than in EWS-FLI1-high EwS cells

We also tested the efficacy of compounds in the EWS-FLI1-low state. Recently, it was shown that EwS cells with lower levels of EWS-FLI1 exhibit enhanced migratory features and are more prone to metastasize [20]. The investigation of drugs which are effective in EWS-FLI1-low cells is therefore of interest for combinatorial approaches to prevent metastasis formation in EwS and to circumvent resistance to EWS-FLI1 targeted therapy due to EWS-FLI1-low EwS cells. For validation of the test system, we confirmed differential sensitivity of EwS cells to the EWS-FLI1 targeting compound YK-2-479 under EWS-FLI1-high versus -low conditions (Supplementary Figure 1B). From our screen, eight compounds fulfilled the hit criteria in the EWS-FLI1-low state, among them one FDA approved anti-dopaminergic drug (droperidol), three drug-like agents (oleandrin, navitoclax and AT9283) and four other chemical entities (endothal, endosulfan, 4-nonyphenol and pseudobaptogenin). We did not identify any exclusive or overrepresented targets in the list of the eight compounds in comparison to all the compounds tested.

### Treatment with BCL-2 inhibitors has opposite effects under EWS-FLI1-high and -low conditions

**Figure 3:**
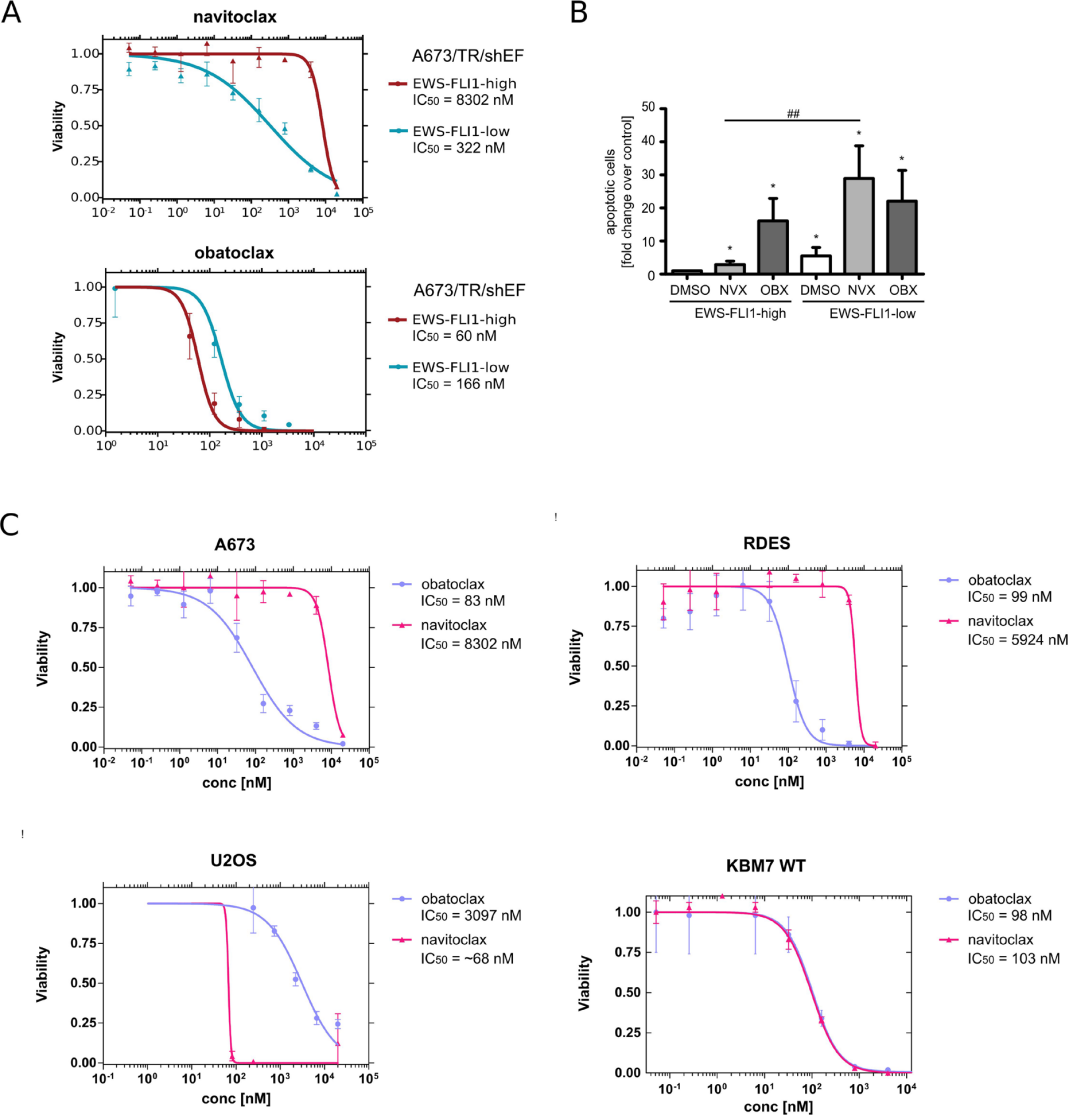
Differential effect of navitoclax and obatoclax on EWS-FLI1-high and -low EwS cells (**A**) Dose response curves of obatoclax and navitoclax in EWS-FLI1 high and low conditions. (**B**) A673/TR/shEF cells were treated with dox for 96 h in total. Navitoclax (3.5 µM) and obatoclax (100 nM) were applied for 72 h. DMSO was used as control. AnnexinV staining was performed, the percentage of apoptotic cells determined, normalized to DMSO control and displayed as relative fold change. Data are shown as means ± SD from 4 independent experiments. (^*^p < 0.05, ^**^p < 0.01, ^***^p < 0.001 - compared to DMSO; ^#^*p <* 0.05, ^##^*p <* 0.01, ^###^*p <* 0.001 NVX treatment compared in EWS-FLI1 expressing versus EWS-FLI1 knockdown cells.) NVX, navitoclax; OBX, obatoclax. (**C**) Upper panel: comparison of the effect between navitoclax and obatoclax in two EwS cell lines (A673 and RDES); lower panel: potency of navitoclax and obatoclax in two non-EwS cell lines (U2OS and KBM7). The pattern of sensitivity for the two drugs was found to be similar in the EwS cell lines, while it differed in the non-EwS cell lines.

Among the small number of compounds that exhibited higher efficacy in EWS-FLI1-low as compared to EWS-FLI1-high EwS cells, was the BCL-2 family inhibitor navitoclax (ABT-263). Interestingly, navitoclax showed an inverse effect to the pan-BCL-2 family inhibitor obatoclax (GX15-070), which is more potent in EWS-FLI1-high EwS cells. Thus, we decided to use the differential activity of the two BCL2 family inhibitors as a model to validate our *in silico* approach. Cell viability in EWS-FLI1-high and -low EwS cells was measured after 72 hours of drug treatment using CellTiter Glo. As demonstrated by our *in silico* analysis we were able to confirm a strong increase in sensitivity of EWS-FLI1-low cells as compared to EWS-FLI1-high cells to navitoclax treatment (Figure 3A). The response of EWS-FLI1-high and -low EwS cells to treatment with obatoclax was comparable, in line with the *in silico* analysis, with a slight increase in EWS-FLI1-high cells. Next, we confirmed that the loss of tumor cell viability in response to navitoclax and obatoclax treatment was due to apoptosis (Figure 3B). While both EWS-FLI1-high and -low cells became apoptotic upon treatment with obatoclax, treatment with navitoclax led to significantly increased apoptosis in EWS-FLI1-low as compared to EWS-FLI1-high cells.

Similar to A673/TR/sh and parental A673 cells, RDES EwS cells used for validation were sensitive to treatment with the pan-BCL-2 inhibitor obatoclax, but resistant to navitoclax (Figure 3C). Conversely, when we compared potencies of the two BCL-2 inhibitors in EWS-FLI1 negative cancer cells, U2OS osteosarcoma and KBM7 chronic myeloid leukemia cell lines, we observed markedly different patterns than in EwS. In contrast to A673 and RDES, in U2OS navitoclax was dramatically more potent than obatoclax, while in KBM7 cells the responses to the two drugs were almost identical (Figure 3C). Navitoclax shares its target spectrum with another BCL-2 small molecule inhibitor, ABT-737 [8, 21]. Consistently, ABT-737 showed a comparable effectiveness in EWS-FLI1-low cells, while EWS-FLI1-high cells were resistant to treatment with ABT-737 (Supplementary Figure 1C). In U2OS, both navitoclax and ABT-737 were more potent at inducing cell death than obatoclax (Supplementary Figure 1D).

### EWS-FLI1 differentially regulates members of the BCL-2 family

Next, we studied the mechanism behind the EWS-FLI1 dependent differential response to the tested BCL-2 family inhibitors. Although the target profiles of obatoclax and navitoclax were similar, affinities and the selectivity for the anti-apoptotic BCL-2 family members differed dramatically [22]. Navitoclax, like ABT-737, shows strong affinity for BCL-2, BCL-X(L), and BCL-W, while they are inactive towards MCL-1 and BCL-B. In contrast, obatoclax targets all BCL-2 family members with similar affinity [22], however, with weaker potency than navitoclax. Next, we tested inhibitors of BCL2, BCL-X(L), and MCL-1 (ABT-199, A-1155463, S63845, respectively). We observed that curve shifts depicting difference in viability between EWS-FLI1-high and -low states upon treatment with either BCL-2 or BCL-X(L) inhibitor resemble the pattern of navitoclax (higher potency in EWS-FLI1-low state), while in contrast the MCL-1 inhibitor was more potent in EWS-FLI1-high state, thus belonging to the larger group of compounds with higher efficacy in EWS-FLI1-high condition (Supplementary Figure 2). Although the shifts are mild, they are in concordance with reported targets of navitoclax and obatoclax and with the phonotypes that we observed.

**Figure 4:**
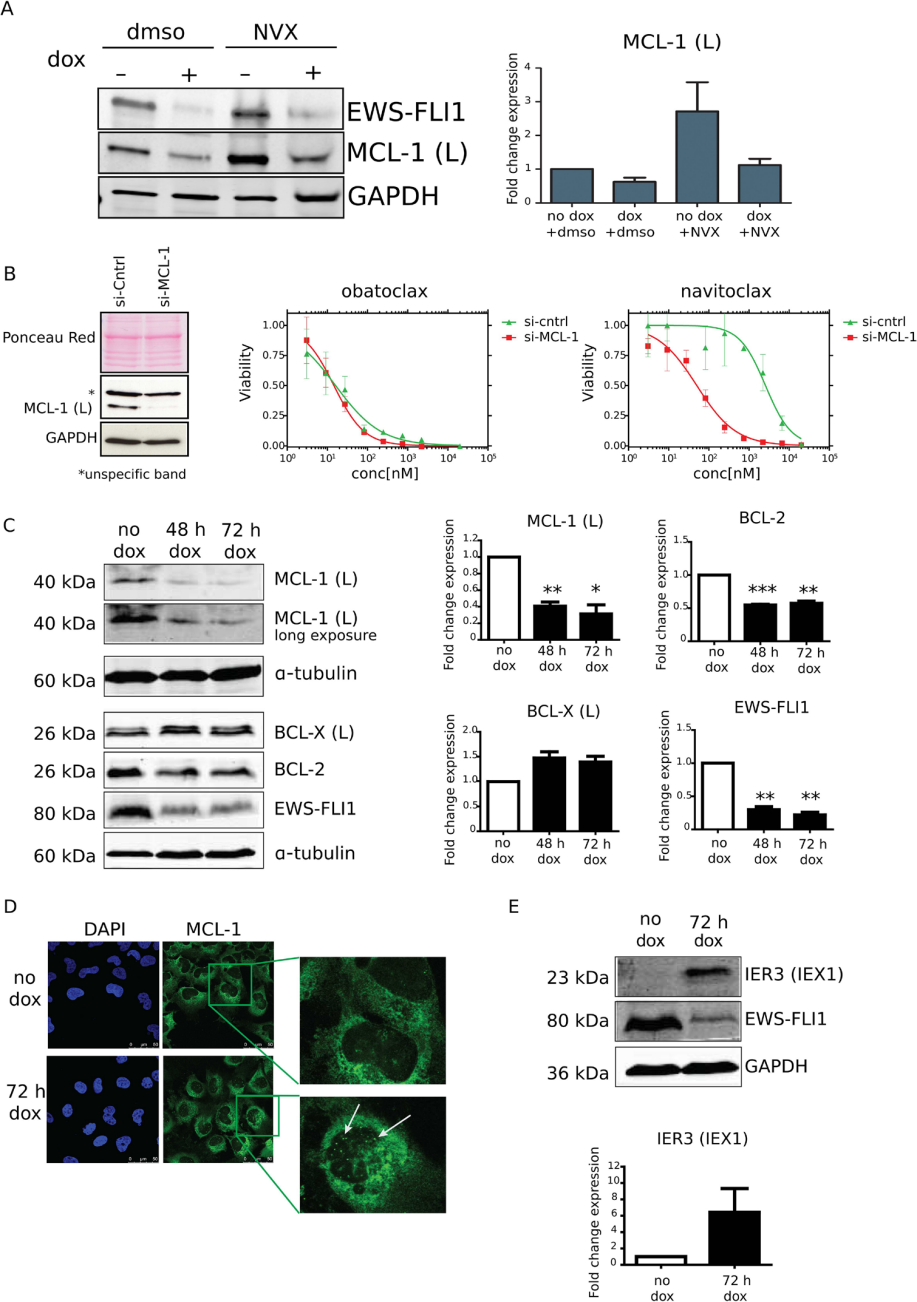
MCL-1 expression levels are EWS-FLI1 dependent and contribute to the drug-induced phenotype (**A**) Immunoblot showing MCL-1 expression upon navitoclax (NVX; 3.5 µM) treatment in A673/TR/shEF. Expression levels of MCL-1 are increased by NVX, unless EWS-FLI1 levels are low (dox). Blot shows quantification of MCL-1 fold change expression relative to GAPDH of two independent experiments. (**B**) Left, knockdown efficiency of MCL-1 upon transcfection with si-MCL-1 or si-control in A673/TR/shEF cells. Right, dose response curves of obatoclax and navitoclax in si-control transfected A673/TR/shEF cell line and upon knockdown of MCL-1. (**C**) Immunoblot image of MCL-1 (L), BCL-X (L) and BCL-2 expression changes upon dox-induced EWS-FLI1 knockdown in A673/TR/shEF EwS cells (48 h and 72 h timepoints) (left) and quantification of protein expression relative to α-TUBULIN from three independent experiments (mean ± SD). *P*-values were calculated using One-Sample *T*-Test comparing expression changes after dox treatment to the untreated control (^*^=≤0.05; ^**^=≤0.01; ^***^=≤0.001) (right). (**D**) Subcellular localization of MCL-1 is shown with immunofluorescence confocal microscopy at 63X magnification, demonstrating the appearance of MCL-1 in nuclear speckles (indicated with arrows) in EWS-FLI1-low cells (dox). One representative experiment from three biological replicates is shown. (**E**) Protein expression of IER3 (IEX1) is highly upregulated in EWS-FLI1-low cells (72 h dox) as shown by western blot. Respective quantification of IER3 bands from three independent experiments was performed by normalization to GAPDH.

Since RNA expression data in several EwS cell lines suggested EWS-FLI1-dependent regulation of MCL-1 expression [10, 23] we hypothesized that MCL-1 levels could explain the differential navitoclax sensitivity in EWS-FLI1-high and -low conditions. Using immunoblot we confirmed a decrease in MCL-1 protein expression upon dox-induced knockdown of EWS-FLI1 in A673/TR/shEF cells (Figure 4A and 4C). Interestingly, treatment with navitoclax increased MCL-1 expression levels strongly in the EWS-FLI1-high, but not -low state (Figure 4A). Upregulation of MCL-1 expression upon treatment with navitoclax or ABT737 has been previously reported as a mechanism of resistance in various cancer cells [9, 24]. To test whether a decrease in MCL-1 expression enhances sensitivity to navitoclax in EWS-FLI1-high EwS cells, we performed a transient knockdown of MCL-1 using siRNA. Strikingly, knockdown of MCL-1 (Figure 4B) led to a similar drug effect on EwS cell viability as the knockdown of EWS-FLI1 in the A673/TR/shEF system. While no changes in sensitivity towards obatoclax were observed, a large shift towards improved sensitivity was noted upon navitoclax treatment. EWS-FLI1 knockdown strongly reduced MCL-1 protein expression (60% reduction) (Figure 4A and 4C), while levels of anti-apoptotic proteins BCL-2 and BCL-X(L) were decreased and increased by 50%, respectively (Figure 4C). We also observed sequestration of MCL-1 to nuclear speckles in the EWS-FLI1-low state, as compared to EWS-FLI1-high (Figure 4D). This pattern is highly reminiscent of the product of the immediate early gene X1 (IEX-1/IER3), which was previously reported to sequester MCL-1 in response to DNA damage [25], at least partially co-localizing with PML bodies in HeLa cervical carcinoma cells [26]. In this cell line, upregulation of IER3 was required for DNA damage to induce apoptosis [27]. Intriguingly, we found IER3 to be strongly induced upon conditional EWS-FLI1 knockdown in A673/TR/shEF (Figure 4E) and, with the only exception of SK-N-MC, in four of five additional cell lines upon transient knockdown of EWS-FLI1 [23]. Ectopic expression of IER3 on its own slightly increased navitoclax sensitivity of A673/TR/shEF cells, though the level of this increase did not achieve significance (Supplementary Figure 3). Thus, it is conceivable that a combination of MCL-1 expression modulation and IER3 dependent subcellular re-localization in response to EWS-FLI1 depletion is contributing to the observed gain in navitoclax sensitivity, suggesting a major role for MCL-1 in apoptosis regulation and navitoclax resistance of EwS.

In the next step we used the near-haploid KBM7 cell line [28] to further investigate the mode of action of BCL-2 inhibitors. Anti-apoptotic BCL-2 proteins function by segregating particular BH3 proteins. Caused by apoptotic stimuli, BH3 sensitizers such as NOXA or BAD liberate the so-called BH3 activators, allowing them to activate membrane permeabilizing proteins BAK and BAX which oligomerize [22, 29], ultimately leading to apoptosis. Thus, pro-apoptotic BH3-only members BAX and NOXA are crucial for the classical intrinsic apoptosis pathway that is regulated by pro- and anti-apoptotic BCL-2 family proteins. Isogenic knockout systems for BAX and NOXA in the KBM7 cell line confirmed different modes of action of navitoclax and obatoclax. While the potency of navitoclax decreased in the absence of either BAX or NOXA, obatoclax remained equally potent, indicating a distinct mechanism of action (Supplementary Figure 4).

## DISCUSSION

We have developed and applied a network-based systems biology approach to investigate aspects of EWS-FLI1 function in the pathogenesis of EwS and identify compounds interfering with EWS-FLI1 driven cell viability.

By performing high-throughput perturbation screens with more than 3000 compounds in an EwS model cell line upon conditional EWS-FLI1 expression (EWS-FLI1-high versus -low), we unraveled novel EWS-FLI1-dependent vulnerabilities. Integrative network analysis of EWS-FLI1 target spectra revealed crucial cellular processes affected, such as translation, histone deacetylation, topoisomerase activity and microtubule organization. We identified 70 compounds with enhanced efficacy exclusively upon high EWS-FLI1 expression. Apart from compounds already included in the backbone of EwS standard therapy protocols (vincristine, doxorubicin) or used in the treatment of refractory disease (e.g. topotecan [30] and irinotecan [31]), we identified novel targeted small molecules which are either used in the treatment of other cancer types, such are obatoclax and belinostat, or already in early clinical development for pediatric indications and therefore hold promise for future use in the treatment of Ewing sarcoma. Among them taxol (paclitaxel) is currently in Phase 1 and 2 clinical trials for several pediatric malignancies. Importantly, it is in a Phase 2 for Ewing Sarcoma (NCT01962103). It is a microtubule-stabilizing drug, but interestingly it also induces apoptosis by binding to and blocking the function of the apoptosis inhibitor protein BCL-2. Topotecan, a semi-synthetic derivative of camptothecin, is a topoisomerase-I inhibitor currently in several pediatric clinical trials, where its efficacy is tested in combination with a number of chemotherapeutics. Gemcitabine, an antimetabolite nucleoside, is also already in clinical trials for pediatric application. It is tested with taxol in pediatric non-central nervous system (CNS) solid tumors (NCT03507491). Bortezomib, a proteasome inhibitor, is tested in combination with the HDAC inhibitor vorinostat to treat different types of solid tumors in children (NCT01132911). Mebendazole is already approved to treat parasitic infections. It is in Phase 1 trial for recurrent pediatric brain cancers that include medulloblastoma and high-grade glioma refractory to standard-of-care therapies (NCT026442919). The HDAC inhibitor Vorinostat (SAHA), although so far only FDA approved for cancer treatment in adults but not yet in children, is in several clinical trials for pediatric patients for example in combination with bortezomib (Phase 1, NCT01132911) or lenalidomide (Phase 1, NCT03050450) for the treatment of high grade or progressive central nervous system tumors, and with etoposide (Phase 2, NCT01294670) for relapsed and treatment refractory sarcomas.

While apoptotic pathways are successfully targeted in multiple cancers, in EwS the cross talk between EWS-FLI1 and apoptosis is less studied. It has been reported that protection from DNA-damage induced apoptosis by EWS-FLI1 and FLI1 involves recruitment of the CBP/P300 coactivator in a transcription independent way [32]. Other studies suggested that EWS-FLI1 binds to the anti-apoptotic protein BCL-2 [33]. Most recently, it was reported that malignant transformation of bone progenitor cells in a transgenic EWS-FLI1 mouse model required expression of MCL1 or other anti-apoptotic BCL-2 family members [34]. Zöllner *et al.* reported that treatment of EwS cells with the EWS-FLI1 inhibiting molecule YK-4-279 induces cell cycle arrest but furthermore supports apoptosis via decreasing anti-apoptotic MCL-1(L) and increasing the pro-apoptotic isoforms of MCL-1(S) and BCL-2 [7]. Our results show that EWS-FLI1 expression potentiates the inhibitory effect of agents like cytarabine, bortezomib and gambogic acid. Most of them induce sensitivity in EwS cells likely through targeting different members of the BCL-2 family. Two well established BCL-2 family inhibitors (obatoclax and navitoclax) were found to have dramatically different effects dependent on EWS-FLI1, suggesting a cross talk between EWS-FLI1 and specific members of the apoptosis regulatory family that are differentially targeted by both drugs. Here, we demonstrate that this crosstalk is mediated via EWS-FLI1 dependent MCL-1 protein expression and conceivably also subcellular re-localization of MCL-1. Our previous ChIP-seq studies identified EWS-FLI1 binding to the MCL-1 promoter [10, 18]. Additionally, we observed that knockdown of EWS-FLI1 greatly reduces the expression of MCL-1, identifying this BCL-2 family member as a directly EWS-FLI1 activated target. We found that EWS-FLI1 modulation results in upregulation of IER3, which was previously reported to be responsible for MCL-1 translocation to nuclear speckles [25]. The appearance of MCL-1 in nuclear speckles was also observed in our EwS model cell line upon knockdown of EWS-FLI1. In 293T cells, IER3 ectopic expression induced apoptosis in an MCL-1 and BIM1 dependent but NOXA and PUMA independent manner [35]. In our study, we found that upregulation of IER3 alone is not sufficient to explain the dramatic gain in navitoclax sensitivity of EwS under EWS-FLI1 low conditions. However, the combination of dampened MCL-1 expression with its altered subcellular localization largely contributes to the apoptotic response to this drug.

To test dependency on individual BCL-2 family members we used siRNA pools and against BCL2, BCL-X(L), and MCL-1 individually and in combination for their influence on viability in A673/TR/sh cells under EWS-FLI1-high and -low conditions (Supplementary Figure 5). They show that genetically inhibiting each of the BCL2 family members individually and in combination results in decreased EwS viability confirming the importance of these molecules for EwS pathogenesis.

It should be noted that, with regard to navitoclax sensitivity, SK-N-MC EwS cells behaved differently from all other EwS cell lines tested. Modulating EWS-FLI1 expression in a dox-inducible EWS-FLI1 shRNA expressing clone of that cell line, shSK-E17T, failed to induce navitoclax sensitivity (Supplementary Figure 6A). Consistent with this finding, EWS-FLI1 knockdown did not reduce MCL-1 nor increase IER3 expression levels (Supplementary Figure 6A and 6B).

Experiments in isogenic near-haploid cell lines lacking different pro-apoptotic BH3-only BCL-2 family members showed that navitoclax activity requires the expression of NOXA or BAX, which was not the case for obatoclax. These findings in EwS cells are consistent with previous results obtained in leukemic cells, where resistance to the navitoclax related BCL-2 inhibitor ABT-737 was demonstrated to be dependent on MCL-1 phosphorylation [36]. Interestingly, it has been reported that the resistance towards ABT-737 could be overcome by increased expression of NOXA, identifying NOXA and MCL-1 as determinants for cell death in ABT-737-resistant cells caused by gossypol, another BCL-2 inhibitor targeting MCL-1 [37]. Although all our data point to an important role of MCL-1 in differential sensitivities of EwS cell lines towards the two BCL-2 inhibitors, it cannot be excluded that additional, so far unknown, targets of obatoclax may also be involved. The targets may not necessarily be related to the inhibition of BCL-2 pro-apoptotic proteins or interfering with intrinsic apoptosis pathways [38–40].

In conclusion, our integrative drug screening analysis methodology in EwS confirmed known EWS-FLI1 interfering mechanisms and discovered novel ones. Moreover, we present a collection of promising small molecule candidates for further pre-clinical development in EwS. The vulnerable EwS biological processes identified in this study provide a basis for future functional investigations and drug synergy screens. In addition, we provide a list of compounds not effective in any of the conditions tested but also a list of compounds effective in both EWS-FLI1-high and -low cells that could inform and accelerate the progress of future EwS therapeutic strategies. Our systems biology approach can be extended and applied in a variety of large-scale compound screen studies tested for multiple conditions and diseases to enable identification of potential drug targets and understand their mechanism of action.

## MATERIALS AND METHODS

### Cell lines and cell culture

All cell lines used in this study are STR authenticated and regularly tested for absence of mycoplasma infections (Mykoalert kit, Lonza, Basel, Switzerland). The EwS model cell line A673/TR/shEF [41] was grown in DMEM (high glucose 4.5 g/l + GlutaMAX, Gibco, Waltham MA USA) supplemented with 10% Tet system Approved Fetal Bovine Serum (Clontech, penicillin and streptomycin, 50 µg/ml Zeocin (Invitrogen, Thermo Fisher; Waltham, MA, USA) and 2 µg/ml blasticidine S hydrochloride (Sigma-Aldrich; St. Louis, MO, USA). To induce the expression of the EWS-FLI1 shRNA, cells were exposed to 1 µg/ml doxycyline (Sigma-Aldrich; St. Louis, MO, USA) for 72 hours. Drug screens in this cell line were initiated 24 h after dox-induced shRNA induction.

The shSKE-17T cell line, a clone of the SK-N-MC EwS cell line with dox-inducible EWS-FLI1 shRNA expression, was kindly provided by O. Delattre (Institut Curie, Paris, France) [20]. For the dose-response curves shown in Supplementary Figure 5, cells were treated for 48 h with dox (1 µg/ml) to induce EWS-FLI1 knockdown and then were treated with Navitoclax (3.5 µM) for 72 h in the presence or absence of dox. Cell lines A673, RDES and U2OS were purchased from the American Type Culture Collection (ATCC). KBM7 cells were grown in Iscove’s modified Dulbecco’s medium (IMDM) with 10% fetal calf serum (FCS). BAX and NOXA knockout cell lines were obtained from Haplogen GmbH, where a gene-trap retrovirus was used to inactivate single human genes in KBM7 as described previously [28].

### Transfections and plasmids

To achieve efficient knockdown of MCL-1 (see Figure 4B) a siRNA Smart Pool from Dharmacon (L-004501-00-0005, Dharmacon; Lafayette, CO, USA) was used together with INTERFERin (Polyplus, Illkirch, France) transfection reagent, according to manufacturer instructions.

Other transfections with siRNA were carried out using Oligofectamine reagent (Invitrogen, Groningen, the Netherlands) according to the manufacturer’s instructions. Knockdown of BCL-2, BCL-X(L) and MCL1 (see Supplementary Figure 2) was achieved by using MISSION^®^ esiRNAs from Sigma-Aldrich (Vienna, Austria); esiRLUC was used as a non-targeting control and esiKIF11 as a positive control, as suggested by the manufacturer.

For ectopic overexpression of IER3 (IEX1), the IER3 expression vector and control vector (Iex1-pcDNA3.1 and pcDNA3.1, respectively, supplied by Prof. Heiner Schäfer, Laboratory of Molecular Gastroenterology and Hepatology, Universitätsklinikum Schleswig-Holstein, Kiel, Germany) were employed. Briefly, using the dox-inducible A673/TR/shEF cell line [41], transfections were performed with the Lipofectamine-Plus reagent (Invitrogen, Groningen, the Netherlands) according to the manufacturer’s recommendations. EWS-FLI1 knockdown and cell viability determinations were done as previously reported [42]. Transfections were carried out using Lipofectamine Plus reagent (Invitrogen, Groningen, the Netherlands).

### High-throughput cell viability screens

Before performing the high-throughput compound screening, cell number was titrated to ensure that cell proliferation remained in a linear exponential phase throughout the experiment. The screens were performed in 384-well format using plates containing compound stock solutions, or DMSO as controls. 1,500 cells per well were seeded in 40 µl of culture medium both with and without dox to maintain EWS-FLI1 shRNA expression throughout the screen. The high-throughput compound screen was performed in three parts summarized in Table 1. First, kinase and phosphatase inhibitors present in the Biomol International compound library (Biomol international; L.P. PA, USA) and Multisource Spectrum compounds (MicroSource Discovery Systems, Inc.; Gaylordsville, CT, USA) consisting of known drugs, other bioavailable compounds and natural products were screened. The second screen comprised MicroSource Cancer drugs and a LOPAC compound library (Sigma-Aldrich; St. Louis, MO, USA) consisting of FDA approved drugs and other compounds with pharmacologically relevant structures. Third, Selleck compound library (Selleckchem Chemicals; Houston, TX, USA) containing FDA approved drugs and additional experimental compounds were used. All compounds were tested in at least two different concentrations. Cell viability was measured after three-day incubation with compounds using CellTiter-Glo (CTG) cell viability assay (Promega; Madison, WI, USA) with Envision Plate-reader (PerkinElmer/Wallac; Waltham, MA, USA) according to manufacturer’s instructions. The raw results were normalized using the LOESS-log normalization algorithm as implemented in R (http://www.r-project.org/). The method down-weights outliers on the plate before calculating the LOESS fit. The Loess-fit approach was chosen to be used in this study as it enables discovery of areas on plates where signal intensities are systematically higher or lower than the signals in adjacent wells on a plate. After LOESS correction, data was divided by the median of negative controls and log2-transformed. For each screen, the compounds that were qualified as hits, were the compounds that inhibited cell viability by at least three median absolute deviations (MADs) from the median of all screened wells. To consider that a compound presents increased effect upon high or low EWS-FLI1, we required that there is at least two-fold decrease in the cell viability upon high EWS-FLI1 compared to low EWS-FLI1 and vice versa.

**Table 1:** Summary of compound screens performed in EWS-FLI1-high and -low A673/TR/shEF Ewing sarcoma (EwS) cells

Screen	Compound library	Compounds	Final concentrations
1	Multisource Spectrum	2000	2.5 µM, 250 nM
	Biomol International	84	25 µM, 2.5 µM, 250 nM, 25 nM
2	Sigma LOPAC	1280	2.5 µM, 250 nM
	Microsource Cancer	80	25 µM, 2.5 µM, 250 nM, 25 nM
3	Selleck	522	25 µM, 8.3 µM, 2.8 µM, 926 nM, 308 nM, 103 nM

### Compound protein interaction data preprocessing and integration

All compounds tested were cross referenced with their respective PubChem identifier [43] to avoid ambiguities caused by synonyms used in the different compound libraries. To obtain information on drugs and their targets, we combined data from the publicly available resources ChEMBL version 19 [13] and STITCH version 9.0 [14]. The raw data from ChEMBL were filtered to remove possibly erroneous entries. The filtering pipeline included removing all data without supporting references, as well as records with missing values, protein names or measurement units. Further, we selected only records with half maximal inhibitory concentration (IC_50_) or inhibition constant (k_i_) measurement units below 1 µM on targeting human proteins. In addition, ChEMBL assigns a confidence score to all entries, indicating the experimental source supporting each entry. We included only data assigned with a confidence score of four or higher (nine is the highest), which indicates a biochemical measurement. The information retrieved from STITCH was derived from the “experimental” and “databases” evidence channels. Only data with a STITCH confidence score equal or above 0.8 were included. Additional manual literature curation was performed for the compounds showing effect. Out of the 3,325 compounds tested we were able to retrieve target information for 1,515 compounds.

### Protein functionality analysis

To investigate processes affected by the compounds of interest, we focused on genes that are exclusive targets or overrepresented in the above compound set. Overrepresented targets were defined using the Fisher’s exact test and false discovery rate (FDR) adjusted *p*-value threshold of 0.05. To investigate potentially affected functions reflected by the above genes we used the Gene Enrichment and Functional Annotation tool provided by the DAVID toolkit [15]. The tool implements a modified Fisher’s exact test to determine genes falling into the same functional categories according to their assigned functional ontology terms. We selected to create the sets of potentially functionally related genes, on terms derived by the Gene Ontology “biological process” and “molecular function” information channels [16] as well as the KEGG pathway database [17]. The default parameters were used, with Benjamin-Hochberg *p*-value adjustment (*q*-value) threshold of 0.01.

### Network construction

The protein interaction network was obtained by using the STRING v9.1 database [44]. To ensure high confidence interactions only, protein interactions assigned as experimentally verified were retrieved and a confidence score above 0.8 was required. Network visualization was performed using the Cytoscape platform v3.2.0.

### Proliferation assays

The individual drug effects were determined in proliferation assays using Cell Titer Glo (Promega Inc., Madison WI, USA) as the readout. Cells were plated in 96-well plates and treated with drugs the following day. Serial dilutions in a range between 20 µM and 0.05 µM were applied for 72 h. In the case where the knockdown of EWS-FLI1 was induced, the induction with dox started 24 h prior to the drug treatment and the cells were kept in dox during the drug treatment. IC_50_ values were determined by fitting a dose response curve to the data points using non-linear regression analysis utilizing the GraphPad Prism software (GraphPad Software, Inc, La Jolla, CA, USA).

### Apoptosis analysis by annexinV staining

To examine the effects of navitoclax and obatoclax on cell death, the number of dead and apoptotic cells was measured by flow cytometry analysis after 72 h inhibitor treatment. Adherent and floating cells were analyzed with the AnnexinV Apoptosis Detection Kit APC (eBioscience, San Diego, USA). AnnexinV and DAPI (Sigma-Aldrich) staining were performed according to manufacturer’s instructions and FACS Fortessa and Diva™ (BD Biosciences, CA, USA) software were used for quantification purposes. As positive control, apoptosis was induced via camptothecin (1 µM) for 48 h. Data were analyzed using the unpaired *t*-test with Welch’s correction or with the one-sample *t*-test using the Prism 5 for Windows (version 5.02) statistical software (GraphPad Prism Software Inc.).

### Immunofluorescence and immunoblot analyses

For immunofluorescence microscopy, cells were fixed with 4% para-formaldehyde (PFA) in phosphate buffered saline (PBS) for ten minutes at room temperature. Cells were permeabilized with 0.3%Triton-X in PBS with addition of 5% goat serum (Dako, Vienna, Austria) for 30 minutes at room temperature. Subsequently, the primary rabbit-MCL-1 (ab32087, Abcam, Cambridge, UK) was added at 1:400 dilution in 0.1%Triton-X/1% bovine serum albumin (BSA)-PBS with 1% goat serum over night at 4° C. The secondary goat-anti rabbit Alexa Fluor 488 antibody (A11034, Life Technologies, Sigma-Aldrich) was diluted in 0.1%Triton-X in 2%BSA-PBS with 1% goat serum and added for 30 minutes at room temperature. Cells were mounted with Vectashield mounting medium containing DAPI (Vectorlabs, Peterborough, UK). Immunostainings were visualized at 63× magnification using the Leica TCS SP8 confocal microscope and images were taken using the Leica LAS-AF software (Leica, Wetzlar, Germany).

For immunoblot analysis, total proteins were resolved by 10% or 12.5% SDS-polyacrylamide gel electrophoresis and processed for immunoblotting. Antibodies used were against MCL-1 (^#^4572, Cell Signaling, New England Biolabs, Frankfurt am Main, Germany), BCL-X(L) (ab178844, Abcam, Cambridge, MA, USA), BCL-2 (ab18210, Abcam), BCL-2 (ab18210, Abcam, Cambridge, MA, USA) IEX-1/IER3 (sc-8454, Santa Cruz, Santa Cruz, CA, USA), FLI1 (MBS177100, My Biosource, San Diego, CA, USA), α-TUBULIN (CP06, Calbiochem, Merck, Vienna, Austria), GAPDH (AM4300, Ambion, Thermo Fisher Scientific). Linear protein quantification was performed using the LICOR Odyssey^®^ Infrared Imaging System and ImageJ software [45].

## SUPPLEMENTARY MATERIALS FIGURES AND TABLES














