# Supplementary material for: Identifying the druggable interactome of EWS-FLI1 reveals MCL-1 dependent differential sensitivities of Ewing sarcoma cells to apoptosis inducers
Source: Oncotarget. 2018 Jul 24;9(57):31018–31. doi: 10.18632/oncotarget.25760 (PMC6089552; doi:10.18632/oncotarget.25760)
Supplement: Supplementary file 1 [file oncotarget-09-31018-s001.pdf]

Identifying the druggable interactome of EWS-FLI1 reveals MCL-1 dependent differential sensitivities of Ewing sarcoma cells to apoptosis inducers

SUPPLEMENTARY MATERIALS

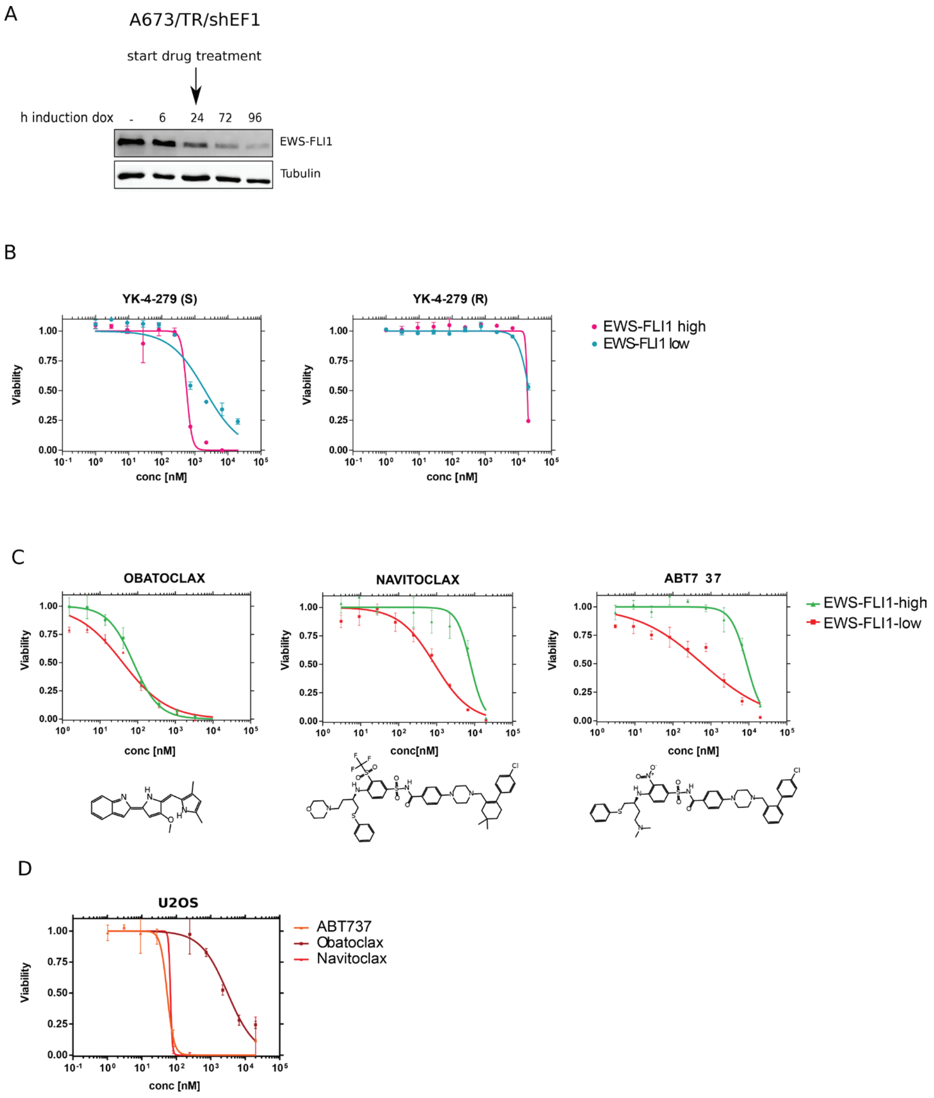

Supplementary Figure 1: Efficacy of YK-2-479, obatoclax, navitoclax in EWS-FLI1-high and –low conditions. (**A**) Immunoblot showing EWS-FLI1 knockdown efficacy. Drug treatments was started 24 h post dox treament for 72 h (total dox treatment time: 96 h). (**B**) Dose response curves of A673/TR/sh cells to YK-2-479 S- (active) and R- (inactive) enantiomers under EWS-FLI1-high and –low conditions. Only the S enantiomer showed efficacy, which was significantly higher in EWS-FLI1-high than –low state. (**C**) Dose response curves of obatoclax, navitoclax and ABT-737 in EWS-FLI1 high and low conditions. Bellow the graph are the corresponding chemical structures. While navitoclax and ABT-737 show the same pattern, obatoclax behaved differently since no curve shift was observed upon the EWS-FLI1 knockdown. (**D**) Dose response curves of the three BCL-2 inhibitors in the osteosarcoma cell line U2OS. Obatoclax is notably less potent than the other two BCL-2 inhibitors, in contrast to ES cells.

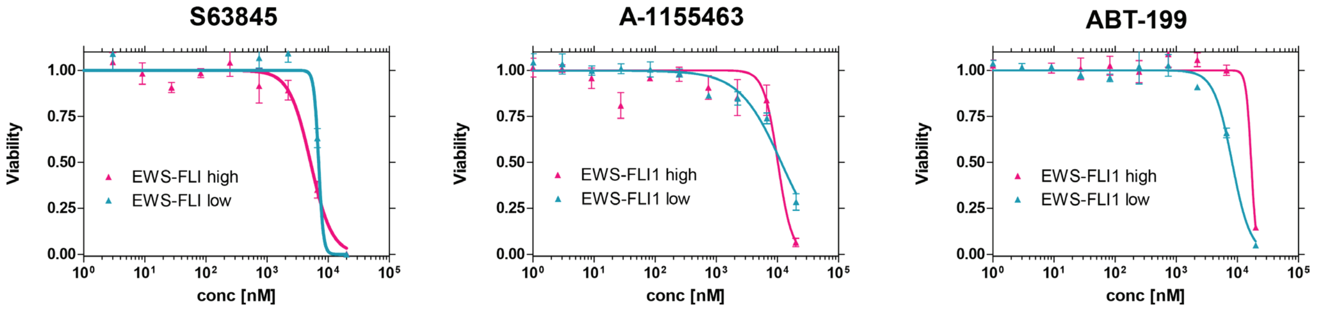

Supplementary Figure 2: Pharmacologic inhibition of BCL-2 family members. Dose response curves of MCL-1, BCL-(X)L and BCL-2 inhibitors (S63845, A-1155463 and ABT-199, respectively) in EWS-FLI1 high and low conditions. A673/TR/shEF cells were treated with drugs for 72 h and cell viability was measured using CellTiter-Glo assay. Curves were fitted according to non-linear regression four-parameter equation in GraphPad Prism.

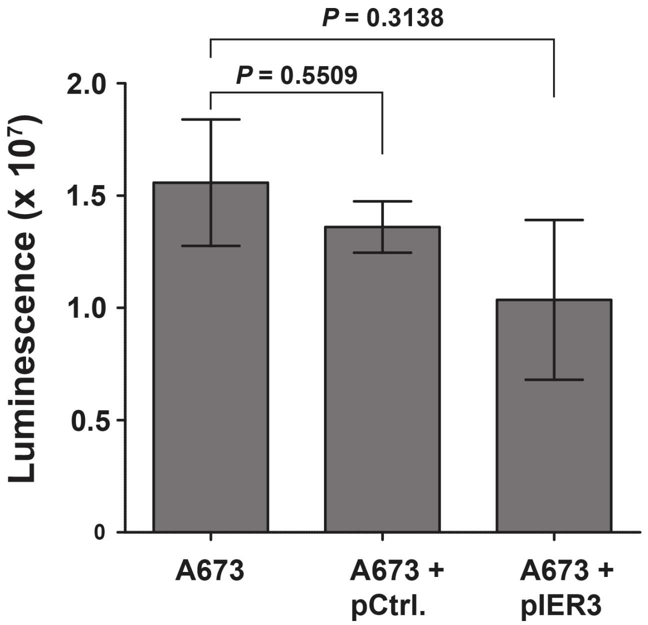

Supplementary Figure 3: Gain of navitoclax sensitivity upon ectopic expression of IER3 in A673/TR/shEF cells. A673/TR/shEF cells were treated with Navitoclax (3,5 μM) and cellular viability (normalized to DMSO control) was measured after 48 h using CellTiter-Glo luminescent cell viability assay. The data are the average of three independent experiments (each performed in triplicates) and are mean ± SD.

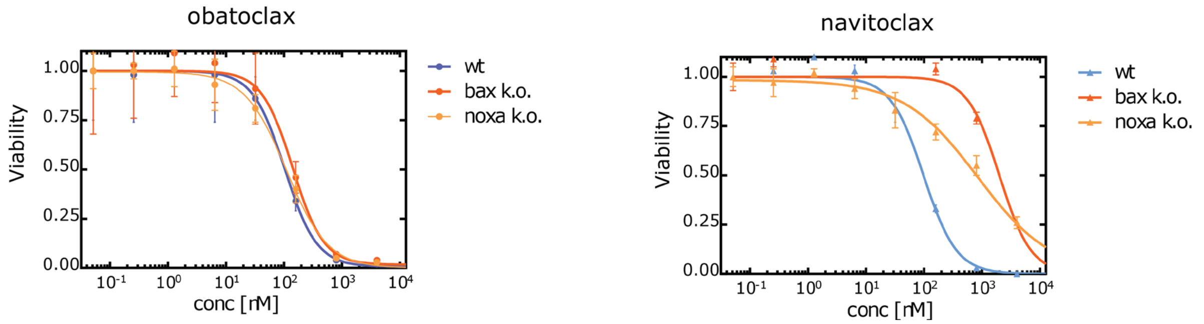

Supplementary Figure 4: Dose response curves of obatoclax and navitoclax in haploid knockout KBM7 clones for BH3-only protein NOXA and membrane permeabilizing proteins BAX, necessary for the intrinsic apoptosis pathway regulated by BCL-2 proteins. Observed are notable shifts in dose response curves of navitoclax, arguing for a classical BCL-2 inhibitor profile. No changes with obatoclax compared to the wild type KBM7 cells.

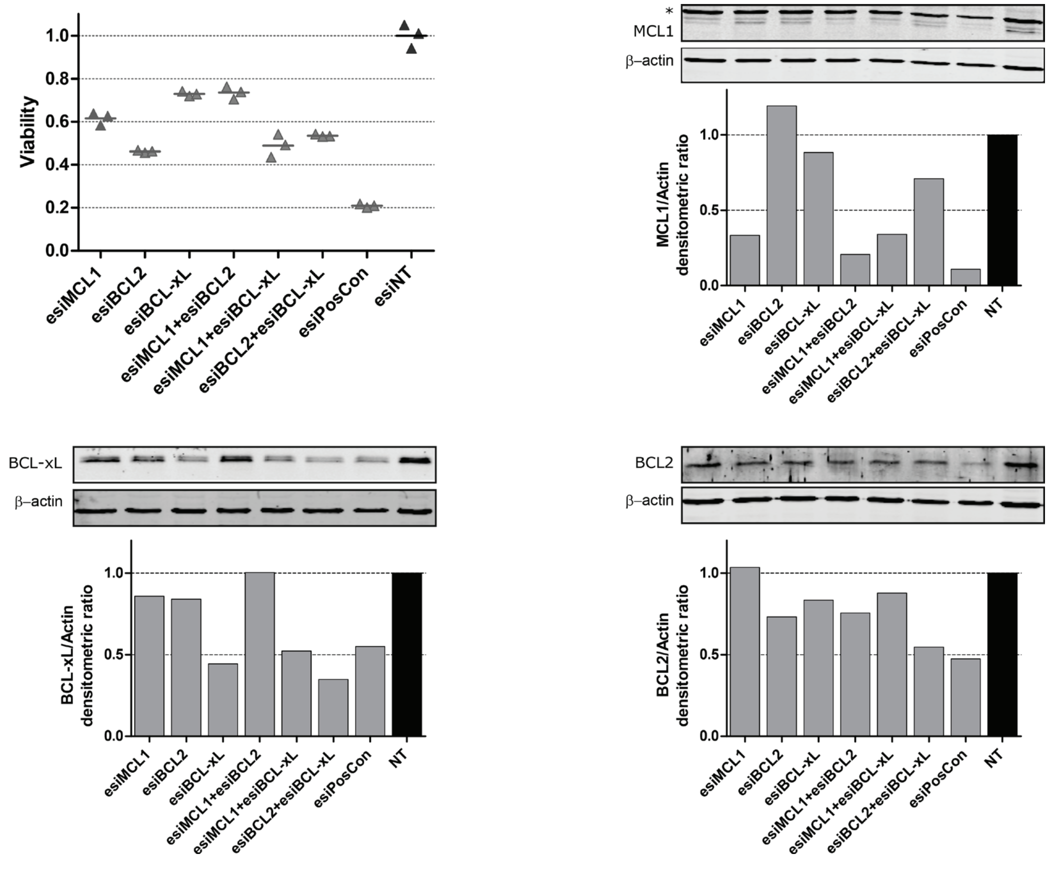

Suplementary Figure 5: Genetic inhibition of BCL2 family members individually and in pairwise combinations. esiRNA mediated knockdown of of MCL-1, BCL-(X)L and BCL-2 in A673/TR/shEF cells. Results are normalized to non-targeting control and one-way ANOVA and Dunnett's multiple comparison test were applied to test for decrease in viability, which showed significance in all tested conditions. Western blots for MCL-1, BCL-(X)L and BCL-2 are shown together with quantification plots.

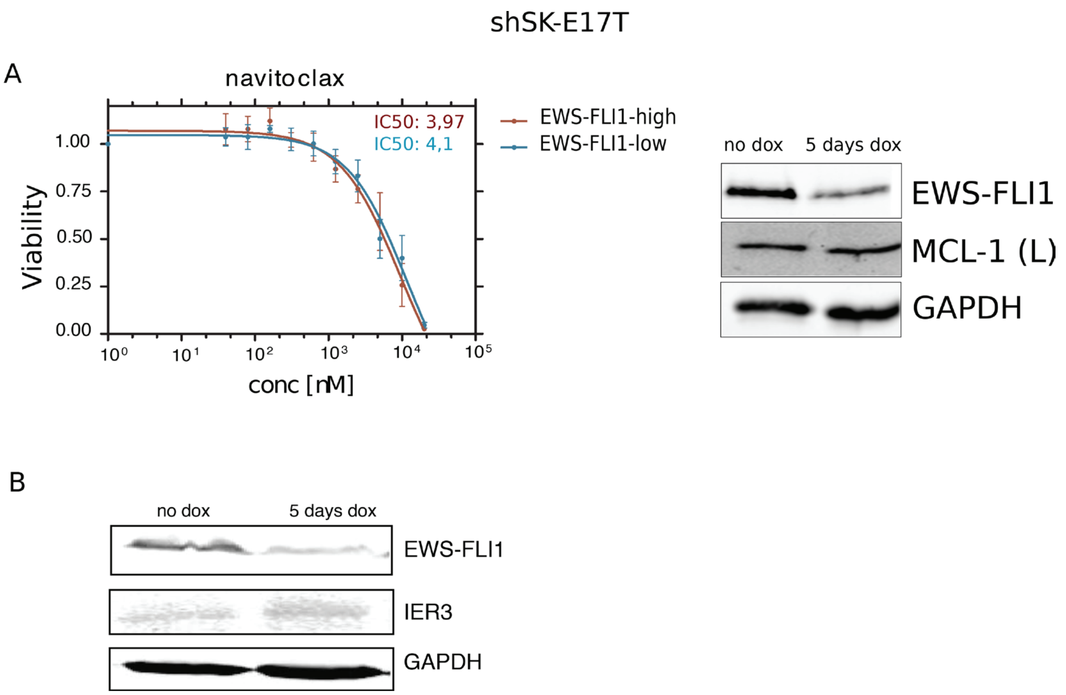

Supplementary Figure 6: Navitoclax dose response and MCL-1 expression of shSK-E17T cells in response to EWS-FLI1 modulation. (**A**) Left: dose response curves in control (IC50: 3,97 uM) and EWS-FLI1 knockdown (IC50: 4,1 uM) shSK-E17T (SK-N-MC) EwS cells. Right: Immunoblot showing knockdown efficacy of EWS-FLI1 in shSKE17T. EWS-FLI1 knockdown was induced by dox treatment 48 h before start of NVX treatment for 72 h (total dox treatment time: 5 days). (**B**) Immunoblot of EWS-FLI1 knockdown in shSK-E17T cells showing IER3 protein expression.

Supplementary Table 1: The complete list of the 3325 compounds tested, screen annotation and the respective cell viabilities measured. See Supplementary_Table_1

Supplementary Table 2: Annotation of compounds tested. See Supplementary_Table_2

Supplementary Table 3: Compounds and reported targets. See Supplementary_Table_3

Supplementary Table 4: List of compound hits upon EWS-FLI1 presence or absence. See Supplementary_Table_4

Supplementary Table 5: Exclusive and enriched targets of the compound hit list. See Supplementary_Table_5

Supplementary Table 6: Biological process, Molecular Function and Pathway Enrichment. See Supplementary_Table_6
